# Impact of repeated cryopreservation on embryo development and chromosomal ploidy

**DOI:** 10.1007/s00404-026-08475-3

**Published:** 2026-06-02

**Authors:** Xi Qin, Li Fan, Yongmei Tang, Wugao Li, Zhetao Li, Yihua Yang

**Affiliations:** 1 Reproductive Medicine Center, Guangzhou Women and Children’s Medical Center Liuzhou Hospital, Liuzhou, 545001 Guangxi China; 2 Reproductive Medicine Center, The First Affiliated Hospital of Guangxi Medical University, Nanning, 530021 Guangxi China; 3 Reproductive Medicine Center, Liuzhou Maternity and Child Healthcare Hospital, Liuzhou, Guangxi China; 4 Guangxi Clinical Research Center for Obstetrics and Gynecology Diseases (GuiKe AD22035223), Liuzhou, 545001 Guangxi China

**Keywords:** Repeated cryopreservation, PGT-A, Euploidy, Pregnancy outcome, Neonatal outcome

## Abstract

**Purpose:**

The potential adverse effects of repeated freezing and thawing processes on embryos remain controversial. This study aimed to investigate the impact of repeated embryo cryopreservation by comparing preimplantation genetic testing for aneuploidy (PGT-A) outcomes and clinical results of single vitrification and double-vitrification embryos.

**Methods:**

This study analyzed the data of 2,335 embryos from 309 patients who underwent PGT-A. Embryos were divided into two groups based on the total number of vitrification procedures: (1) the CV − group (single vitrification, *n* = 1281), where fresh embryos were directly cultured to the blastocyst stage and vitrified once after trophectoderm biopsy, and (2) the CV + group (double vitrification, *n* = 1054), where cleavage-stage embryos underwent an initial vitrification, were subsequently warmed and cultured to the blastocyst stage for biopsy, and then vitrified again.Please check and confirm that the authors and their respective affiliations have been correctly identified and amend if necessary.We confirm that the author list and their respective affiliations have been carefully verified and are correct. We have made minor editorial amendments to the English wording of the institutional names to ensure accuracy and consistency with official nomenclature.

**Results:**

The CV + group exhibited significantly lower blastocyst formation rate (39.6% vs. 46.6%, adjusted difference: − 6.7%, *P* = 0.004), high-quality blastocyst formation rate (11.2% vs. 16.7%, adjusted difference: − 5.3%, *P* = 0.001), and euploid rate per cleavage-stage embryo (7.8% vs. 10.6%, adjusted difference: − 2.6%, *P* = 0.043) than the CV − group. In contrast, the euploid rate per biopsied blastocyst was not significantly different between groups (19.0% vs. 21.9%, *P* = 0.175). Complementary embryo-level mixed-effects logistic regression likewise showed no statistically significant association between double vitrification and embryo euploidy outcomes (adjusted OR 0.72, 95% CI 0.49–1.07; *P* = 0.101). Age-stratified analyses suggested that these detrimental laboratory effects appeared numerically greater in women aged ≥ 38 years, although formal interaction testing did not support statistically significant effect modification by age. No statistically significant differences were observed in live birth or neonatal outcomes following euploid blastocyst transfer; however, these exploratory comparisons were based on limited transfer numbers and, therefore, should be interpreted cautiously.

**Conclusions:**

Repeated vitrification-warming exposure was associated with reduced embryo developmental efficiency and a numerically lower euploid embryo yield per cleavage-stage embryo. No statistically significant differences were observed in reproductive outcomes after euploid blastocyst transfer; however, these analyses were exploratory and substantially underpowered to exclude clinically meaningful differences. Larger adequately powered studies are required to further clarify the reproductive implications of repeated cryopreservation.

**Supplementary Information:**

The online version contains supplementary material available at 10.1007/s00404-026-08475-3.

## What does this study add to the clinical work

**Table:** 

Repeated embryo cryopreservation may reduce the efficiency of obtaining transferable euploid embryos during PGT-A treatment. Clinicians should consider the potential developmental cost of additional vitrification-warming procedures when planning embryo accumulation strategies.	

## Introduction

Embryo cryopreservation has become an integral component of modern in vitro fertilization (IVF) practice. In addition to increasing cumulative live birth opportunities and reducing the risk of ovarian hyperstimulation syndrome (OHSS), it also provides greater flexibility for embryo transfer scheduling. In patients undergoing preimplantation genetic testing (PGT), embryo cryopreservation allows sufficient time for genetic analysis and facilitates synchronization between embryo development and endometrial receptivity during frozen embryo transfer (FET) cycles [1]. In some fertility centers, FET has gradually replaced fresh embryo transfer as the predominant transfer strategy [2].

With the widespread adoption of embryo cryopreservation, clinical scenarios involving repeated freezing and warming procedures have become increasingly common, particularly in PGT cycles. Patients with advanced female age or diminished ovarian reserve may require repeated ovarian stimulation and oocyte retrieval cycles to accumulate sufficient embryos for biopsy [3, 4]. To improve efficiency and reduce cost, some reproductive centers have adopted an “embryo pool” strategy, in which cleavage-stage embryos from multiple cycles are sequentially vitrified and subsequently warmed together for blastocyst culture and biopsy alongside fresh embryos obtained in the final cycle [5]. Consequently, some embryos are exposed to planned repeated vitrification-warming procedures.

Currently, the embryologic and reproductive impact of repeated embryo cryopreservation remains controversial. Although several retrospective studies have investigated this question [6–9], a recent meta-analysis suggested that repeated vitrification may be associated with reduced clinical pregnancy and live birth outcomes after euploid embryo transfer [10]. However, most previous PGT-based studies primarily involved embryo re-biopsy after inconclusive genetic testing results, making it difficult to fully distinguish the independent effects of repeated cryopreservation from those of additional biopsy manipulation. In contrast, the embryo-pooling strategy provides a relatively clearer clinical setting for evaluating repeated vitrification exposure. Therefore, we retrospectively analyzed chromosome screening results, pregnancy outcomes, and neonatal outcomes among patients undergoing this strategy in our center to further investigate the embryologic and reproductive consequences of double vitrification.

## Materials and methods

### Patients

This retrospective study included preimplantation genetic testing for aneuploidy (PGT-A) patients who underwent multiple ovarian stimulation cycles at the Reproductive Center of Guangzhou Women and Children’s Hospital Medical Center Liuzhou Hospital from August 2020 to December 2023. These patients underwent repeated ovarian stimulation and oocyte retrieval, because an insufficient number of embryos were obtained from a single stimulation cycle. Cleavage-stage embryos obtained in previous cycles were vitrified and stored until the final stimulation cycle, after which they were warmed and cultured to the blastocyst stage for biopsy alongside fresh embryos obtained in the final cycle. Indications for PGT-A included advanced maternal age (AMA; ≥ 38 years), recurrent spontaneous abortion (RSA), and recurrent implantation failure (RIF). Patients without embryo vitrification-warming procedures or those who underwent concurrent PGT-M and PGT-A for monogenic disorders were excluded.

**Fig. 1:**
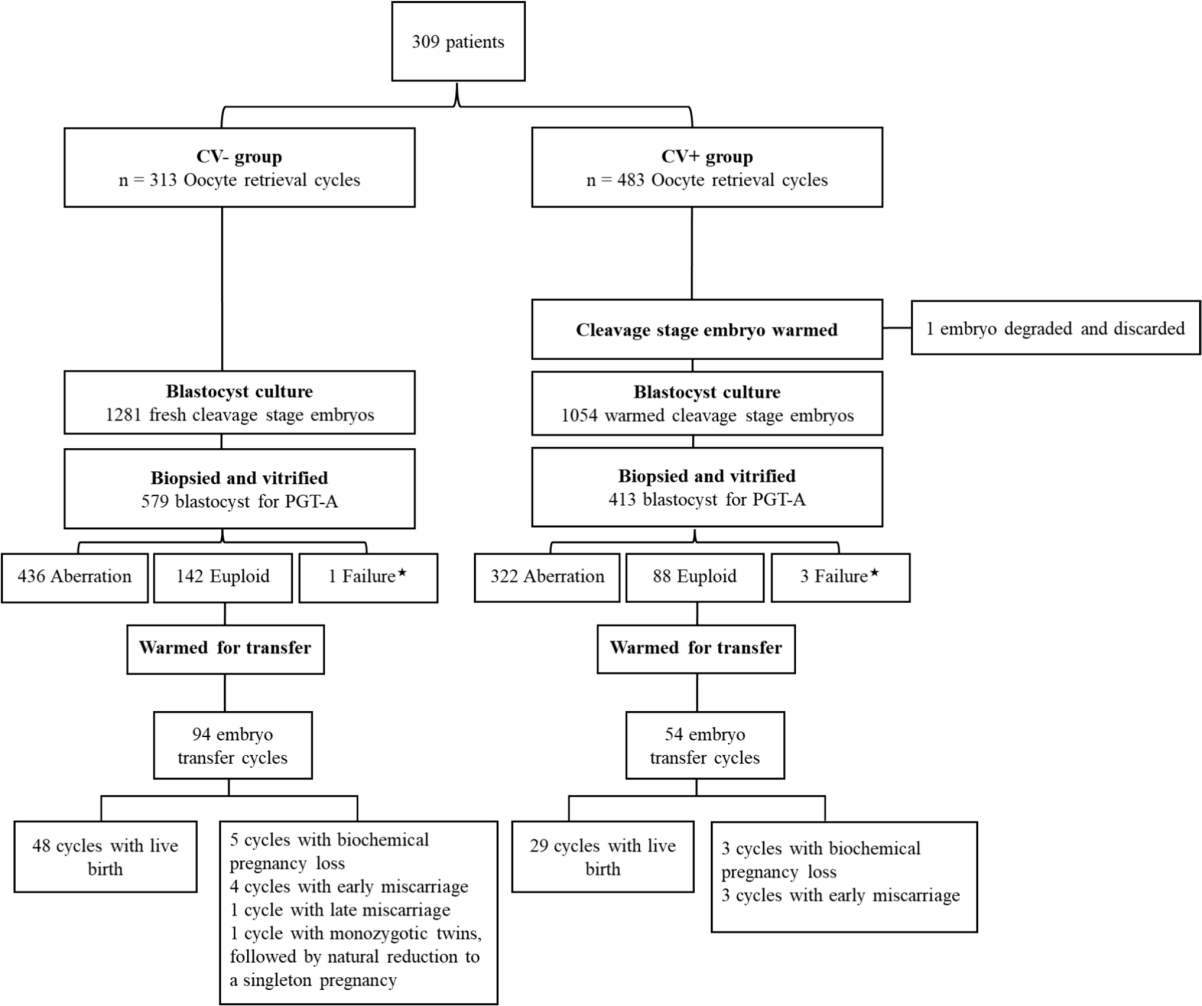
Study flowchart. CV−: single vitrification group (fresh embryos directly cultured to the blastocyst stage); CV + : double-vitrification group (cleavage-stage embryos vitrified, subsequently warmed, and cultured to the blastocyst stage)

Cycles were classified according to whether embryos underwent cleavage-stage vitrification, Fig. 1:

(i) Single vitrification group (CV − group): fresh embryos were directly cultured to the blastocyst stage, underwent trophectoderm biopsy, and were vitrified once after biopsy (*n* = 313 cycles) and (ii) double-vitrification group (CV + group): cleavage-stage embryos were first vitrified and stored until the final cycle, then warmed and cultured to the blastocyst stage for trophectoderm biopsy, followed by repeat vitrification after biopsy, resulting in repeated vitrification-warming exposure (*n* = 483 cycles).

### Embryo culture and vitrification/warming procedure

Ovarian stimulation protocols were selected according to individual ovarian reserve characteristics and included progestin-primed ovarian stimulation (PPOS), antagonist, and other protocols. Oocytes were retrieved 34–36 h after HCG trigger and fertilized by intracytoplasmic sperm injection (ICSI). Fertilization was assessed 16–18 h later. On day 3, cleavage-stage embryos were morphologically evaluated according to the Scott criteria [11]. Blastocysts formed on days 5–7 were graded according to the Gardner scoring system [12], and blastocysts with a morphology score of ≥ 3BB were defined as high quality. A commercial vitrification kit (KITAZATO, Japan) was used for all vitrification and warming procedures according to the manufacturer’s standardized protocol.

On the day of blastocyst formation, trophectoderm biopsy was performed for blastocysts meeting the following criteria: expanded or hatching blastocyst stage, inner cell mass grade ≥ B, and trophectoderm grade ≥ C. Occasionally, 3CC blastocysts were also biopsied based on clinical judgment. Laser-assisted biopsy was used to obtain 5–8 trophectoderm cells for genetic analysis. After biopsy, blastocysts underwent repeat vitrification. Before vitrification, artificial shrinkage was performed using laser collapse. Subsequent vitrification and warming procedures were conducted according to the same standardized protocol used for cleavage-stage embryos.

### Preimplantation genetic testing for aneuploidy

Whole-genome amplification was performed using multiple displacement amplification (MDA) (REPLI-g Single Cell Kit, QIAGEN Inc.). Amplified products were analyzed using a preimplantation embryo chromosome aneuploidy detection kit (Jabrehoo, China), and sequencing was conducted on the MiSeq high-throughput sequencing platform (Illumina, USA). Bioinformatic analysis of raw sequencing data was performed using PGXCloud (Jabrehoo, China). According to the original laboratory reporting framework used during the study period, embryos with mosaic findings were incorporated into the non-euploid/aberrant category rather than recorded as a separate analytic subgroup.

### Blastocyst transfer

Endometrial preparation was performed using natural cycles, ovulation induction cycles, or hormone replacement cycles [13]. A single euploid blastocyst was transferred on day 5 after luteal transformation. Serum human chorionic gonadotropin (HCG) levels were measured 12 days after embryo transfer. Gestational sacs and fetal heartbeats were confirmed by transvaginal ultrasound 30–35 days after transfer.

### Statistics

Statistical analyses were performed using R version 4.5.2 (R Foundation for Statistical Computing, Vienna, Austria). Continuous variables were presented as mean ± standard deviation (SD) or median with interquartile range (IQR) according to data distribution, and compared using Student’s *t* test or the Mann–Whitney *U* test as appropriate. Categorical variables were expressed as frequencies and percentages and compared using the chi-square test or Fisher's exact test. To account for embryos clustered within patients, linear mixed models with cluster-robust standard errors and patient-level random intercepts were used to compare embryologic development and euploidy-related outcomes. Adjusted mean differences with 95% confidence intervals (CIs) were estimated after controlling for female age at oocyte retrieval. Exploratory age-stratified analyses (≥ 38 years vs. < 38 years) were additionally performed using the same modeling framework, and formal group-by-age interaction terms were tested to evaluate potential effect modification. Multivariable mixed-effects logistic regression with a patient-level random intercept was used to identify factors associated with blastocyst euploidy. As complementary embryo-level analyses, generalized linear mixed models with binary euploid vs. non-euploid status as the dependent variable and patient-level random intercepts were additionally fitted to reassess euploid embryo probability. For principal euploidy-related and reproductive endpoints, detectable effect size and minimum detectable-difference analyses were additionally performed to provide a quantitative context for interpreting statistically nonsignificant findings under the observed sample sizes. All tests were two-sided, and *P* < 0.05 was considered statistically significant.

## Results

In this study, the main indication for PGT-A treatment was advanced age, and the main ovarian stimulation protocols used were the PPOS and antagonist protocols. There were no significant differences between the two groups in oocyte maturation rate, normal fertilization rate, normal cleavage rate, and high-quality cleavage-stage embryo rate (Table 1). A total of 1281 cleavage-stage embryos were obtained in the CV − group, all of which underwent blastocyst culture. In the CV + group, 1194 cleavage-stage embryos were obtained. Among them, 139 embryos were either transferred in fresh cycles or discarded because of poor quality before cryopreservation. The remaining 1055 embryos underwent vitrification cryopreservation. After warming, one embryo degenerated and was excluded, leaving 1054 embryos for subsequent blastocyst culture.

**Table 1:** Characteristics and ovarian stimulation outcomes of the CV− and CV + groups

Group	CV − group	CV + group	*P *value
Oocyte retrieval cycles	313	483	
Female age at retrieval (years)	41.00 (39.00,43.00)	41.00 (39.00,43.00)	0.602
AFC	5.00 (4.00,8.00)	5.00 (4.00,7.00)	0.425
basal FSH (IU/L)	6.60 (5.30,8.39)	6.75 (5.39,8.75)	0.474
BMI (kg/m^2^)	22.40 (20.80,24.70)	22.50 (20.80,24.70)	0.651
Indication, *n* (%)			0.647
AMA	182 (58.15)	273 (56.52)	
RSA	110 (35.14)	183 (37.89)	
RIF	21 (6.71)	27 (5.59)	
Protocol, *n* (%)			0.476
PPOS	135 (43.13)	199 (41.20)	
Antagonist	115 (36.74)	169 (34.99)	
Others	63 (20.13)	115 (23.81)	
Total dosage of Gn (IU)	2400.00 (2025.00,2775.00)	2175.00 (1650.00,2550.00)	< 0.001
Duration of stimulation (days)	9.00 (8.00,10.00)	8.00 (7.00,9.00)	< 0.001
MII rate, %	100 (80.00,100.00)	100.00 (72.73,100.00)	0.289
2PN rate, %	80.00 (66.67,100.00)	80.00 (50.00,100.00)	0.320
Normal cleavage rate, %	100 (100.00,100.00)	100 (100.00,100.00)	0.750
High-quality cleavage-stage embryo rate, %	50.00 (22.22;75.00)	50.00 (0.00;100.00)	0.750

Data were presented as median (P25, P75) or numbers (%)

*AMA* Advanced maternal age, *RSA* recurrent spontaneous abortion, *RIF* repeated implantation failure, *PPOS* progestin-primed ovarian stimulation, *BMI* body mass index, *FSH* follicle-stimulating hormone, *Gn* gonadotropin, *MII* metaphase II, *2PN* two pronuclei

**Fig. 2:**
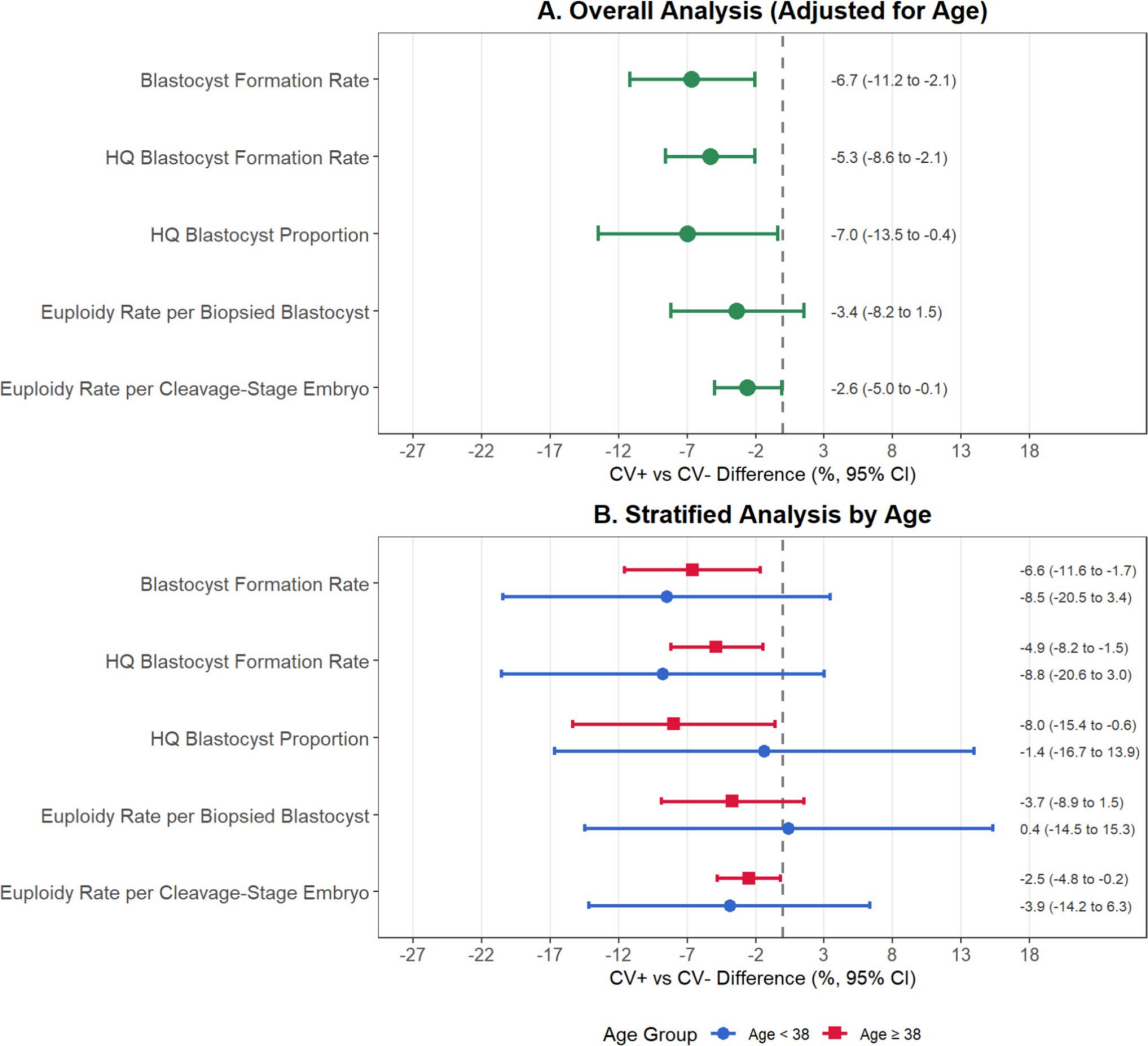
Forest plots comparing embryologic and euploidy-related outcomes between the CV + and CV − groups. *HQ,* high-quality blastocyst. Data are presented as adjusted mean differences with 95% confidence intervals (*CIs*) derived from linear mixed models with cluster-robust standard errors. **A** Overall analysis adjusted for female age at retrieval. **B** Exploratory age-stratified analyses, with blue squares representing women aged < 38 years and red diamonds representing women aged ≥ 38 years. Error bars crossing the vertical reference line indicate no statistically significant between-group difference (*P* ≥ 0.05)

Among biopsied blastocysts, 142 euploid embryos were identified from 579 blastocysts in the CV − group, compared with 88 euploid embryos from 413 biopsied blastocysts in the CV + group. Compared with the CV − group, the CV + group demonstrated significantly lower blastocyst formation rate (39.6% vs. 46.6%, adjusted difference: − 6.7%, *P* = 0.004), significantly lower high-quality blastocyst formation rate (11.2% vs. 16.7%, adjusted difference: − 5.3%, *P* = 0.001), and a numerically lower euploid yield per cleavage-stage embryo (7.8% vs. 10.6%, adjusted difference: − 2.6%, *P* = 0.043). In contrast, no statistically significant difference was observed in euploid rate per biopsied blastocyst after age adjustment (19.0% vs. 21.9%, adjusted difference: − 3.4%, *P* = 0.175) (Table 2). Embryo-weighted crude rates calculated from pooled event counts showed directionally similar between-group patterns across all five laboratory endpoints (Supplementary Table S1). Age-stratified analyses suggested that adverse laboratory trends appeared numerically greater in women aged ≥ 38 years, with statistically significant reductions observed in blastocyst formation, high-quality blastocyst formation rate, and euploid yield per cleavage-stage embryo within this subgroup (Fig. 2). However, formal interaction testing between vitrification group and age stratum did not reach statistical significance for any endpoint (all *P* for interaction > 0.05; Supplementary Table S2).

**Table 2:** Comparison of embryologic and euploidy-related outcomes between the CV − and CV + groups

Outcome	CV- group (Mean ± SD)	CV + group (Mean ± SD)	Crude difference (95% CI)	Crude* P* value	Adjusted difference* (95% CI)	Adjusted *P* value*
Blastocyst formation rate, %	46.6 ± 31.8	39.6 ± 38.6	−7.0 (−11.6 to −2.4)	0.003	−6.7 (−11.2 to −2.1)	0.004
High-quality blastocyst formation rate, %	16.7 ± 24.3	11.2 ± 25.0	−5.5 (−8.7 to −2.3)	0.001	−5.3 (−8.6 to −2.1)	0.001
High-quality blastocyst proportion, %	35.2 ± 39.0	28.4 ± 41.1	−6.8 (−13.3 to −0.3)	0.041	−7.0 (−13.5 to −0.4)	0.037
Euploid rate per biopsied blastocyst, %	21.9 ± 31.2	19.0 ± 35.1	−2.9 (−7.8 to 2.0)	0.248	−3.4 (−8.2 to 1.5)	0.175
Euploid rate per cleavage embryo, %	10.6 ± 18.9	7.8 ± 20.6	−2.7 (−5.2 to −0.3)	0.030	−2.6 (−5.0 to −0.1)	0.043

Data are presented as mean ± standard deviation. Linear mixed models with cluster-robust standard errors were used to account for embryos clustered within patients. Adjusted differences were controlled for female age at retrieval. The reported estimates represent patient-clustered mixed-model-based cycle-level analyses. Embryo-weighted crude descriptive rates derived from pooled event counts are provided separately in Supplementary Table S1 for contextual comparison

Sensitivity analyses restricted to embryos with complete laboratory follow-up yielded directionally consistent findings with the primary analyses (Supplementary Table S3 and Supplementary Figure S1). The same pattern of numerically greater adverse laboratory trends in women aged ≥ 38 years was also observed.

In multivariable generalized linear mixed-effects logistic regression analyses evaluating factors associated with blastocyst euploidy, double vitrification was not independently associated with euploid status after adjustment for female age, blastocyst quality, and other clinically relevant covariates (adjusted OR 0.76, 95% CI 0.50–1.17; *P* = 0.218). Female age and high-quality blastocyst morphology remained significant independent predictors of blastocyst euploidy (Table 3). To further explore embryo-level associations, complementary generalized linear mixed-model analyses treating euploid embryo status as a binary outcome with patient-level random intercepts were additionally performed. These analyses demonstrated numerically lower euploid probabilities in the CV + group; however, the associations did not reach statistical significance in either the full cohort (adjusted OR 0.72, 95% CI 0.49–1.07; *P* = 0.101) or the complete follow-up cohort (adjusted OR 0.69, 95% CI 0.45–1.07; *P* = 0.099) (Supplementary Table S4).

**Table 3:** Multivariable mixed-effects logistic regression analysis of factors associated with blastocyst euploid status

	Adjusted OR (95% CI)	*P *value
Group		
CV− (Ref)	1.00	–
CV +	0.76 (0.50–1.17)	0.218
Female age (per year)	0.85 (0.81–0.90)	< 0.001
Blastocyst quality		
Low-grade (Ref)	1.00	–
High-grade (3BB +)	2.15 (1.46–3.15)	< 0.001
Biopsy day		
Day 5 (Ref)	1.00	–
Days 6/7	0.96 (0.66–1.41)	0.852
Gn stimulation days	1.10 (0.95–1.27)	0.187
Gn total dose	1.00 (0.99–1.00)	0.235
Protocol		
PPOS (Ref)	1.00	–
Antagonist	0.91 (0.61–1.33)	0.614
Others	0.74 (0.48–1.15)	0.181
Indication		
AMA (Ref)	1.00	–
RSA	1.01 (0.68–1.52)	0.949
RIF	1.14 (0.63–2.10)	0.660
Cycle number	0.86 (0.67–1.12)	0.273

High-quality blastocyst (3BB +), blastocyst with a morphology score of ≥ 3BB; *CI* confidence interval, *OR* odds ratio, *ICC* intraclass correlation coefficient

*Model diagnostics: patient-level random intercept variance = 0.133 (*SD* = 0.364); ICC = 3.87%; AIC = 1010.9; no evidence of overdispersion (dispersion ratio = 1.013, *P* = 0.78)

By September 2024, a total of 148 ET cycles had been performed, including 94 in the CV- group and 54 in the CV + group, resulting in 48 and 29 live births, respectively. All blastocysts survived after warming. In exploratory analyses of reproductive outcomes after euploid blastocyst transfer, no statistically significant differences were observed between groups in clinical pregnancy rate (56.38% vs. 59.26%, *P* = 0.733), live birth rate (51.06% vs. 53.70%, *P* = 0.757), or early miscarriage rate (7.50% vs. 9.40%, *P* > 0.999) (Table 4). Neonatal outcomes, including gestational age, birthweight, and birth length, were likewise numerically similar between groups. However, complementary detectable-difference analyses indicated limited statistical power to exclude clinically meaningful reproductive differences between groups under the current transfer sample size (Supplementary Table S5).

**Table 4:** Reproductive and neonatal outcomes after single euploid blastocyst transfer in the CV − and CV + groups

Group	CV − group	CV + group	*P *value
No. of ET cycles	94	54	
Female age at ET (years)	39.00 (38.00,41.00)	39.50 (37.25,41.00)	0.646
Indication			0.566
AMA, *n* (%)	43 (45.74)	23 (42.59)	
RSA, *n* (%)	38 (40.43)	26 (48.15)	
RIF, *n* (%)	13 (13.83)	5 (9.26)	
Endometrial preparation			0.417
Natural cycle, *n* (%)	21 (22.34)	17 (31.48)	
Ovarian stimulation, *n* (%)	26 (27.66)	15 (27.78)	
Hormone replacement, *n* (%)	47 (50.00)	22 (40.74)	
Endometrial thickness (mm)	9.00 (8.20,11.00)	9.50 (8.70,10.30)	0.236
High-quality blastocyst formation rate, *n* (%)	54/94 (57.45)	25/54 (46.30)	0.191
Biochemical pregnancy rate, *n* (%)	58/94 (61.70)	35/54 (64.81)	0.706
Clinical pregnancy rate, *n* (%)	53/94 (56.38)	32/54 (59.26)	0.733
Live birth rate, *n* (%)	48/94 (51.06)	29/54 (53.70)	0.757
Early miscarriage rate, *n* (%)	4/53 (7.50)	3/32 (9.40)	> 0.999*
Neonatal outcomes			
Gestational age (days)	273.00 (266.00,277.00)	270.00 (264.00,274.00)	0.145
Birthweight (g)	3208.85 ± 431.25	3111.72 ± 390.97	0.254
Neonatal length (cm)	50.00 (49.00,51.00)	50.00 (49.00,51.00)	0.957

Biochemical pregnancy was defined as serum HCG ≥ 25 mIU/mL at 12 days after embryo transfer (*ET*). Clinical pregnancy was defined as the presence of a gestational sac on ultrasound examination 30–35 days after ET. Early miscarriage was defined as pregnancy loss before 14 gestational weeks after clinical pregnancy confirmation. Live birth was defined as the delivery of at least one live-born infant after 24 gestational weeks

*Fisher’s exact test was used where appropriate

## Discussion

In this study, we comprehensively evaluated the impact of repeated cryopreservation on embryo developmental competence, chromosomal screening outcomes, and subsequent transfer results. Overall, an additional freeze–thaw procedure at the cleavage stage was associated with statistically significant reductions in blastocyst formation, high-quality blastocyst generation, and euploid embryo yield calculated from the original cleavage-stage embryo cohort. In contrast, no statistically significant difference was observed in the detectable euploid rate per biopsied blastocyst. Likewise, no statistically significant differences were detected in pregnancy or neonatal outcomes after single euploid blastocyst transfer; however, these downstream reproductive comparisons were based on relatively limited transfer numbers and, therefore, should be interpreted cautiously.

In clinical practice, repeated embryo cryopreservation is no longer uncommon because of various scenarios, such as survival of surplus embryos after warming, unplanned genetic testing, or the need for repeat biopsy. Nevertheless, available evidence remains limited and inconsistent [10]. Li et al. [14] reported that blastocyst re-vitrification following prior cleavage-stage vitrification significantly reduced implantation and live birth rates. Their accompanying mouse embryo experiments further demonstrated increased Caspase-3 expression after repeated vitrification, suggesting a cumulative cryoinjury effect, particularly when re-vitrification occurs after morula-stage development. However, that study excluded PGT cycles and, therefore, could not evaluate the impact of repeated cryopreservation on embryo chromosomal constitution. In contrast, several studies in PGT settings suggested that acceptable transfer outcomes may still be achievable after double vitrification [15, 16]. A subsequent small-sample study from the same research group also observed reduced blastocyst formation and high-quality blastocyst rates in thawed cleavage-stage embryos, whereas post-transfer pregnancy outcomes did not significantly differ [17]. However, the inclusion of heterogeneous PGT indications and biopsy strategies may have introduced additional confounding.

Using a relatively homogeneous PGT-A cohort and a standardized embryo-pooling workflow, this study similarly demonstrated that embryos exposed to repeated vitrification exhibited reduced developmental efficiency compared with embryos undergoing only a single vitrification procedure. Previous experimental and clinical studies have suggested that cryopreservation may affect embryos through alterations in cellular ultrastructure, mitochondrial function, metabolic regulation, cytoskeletal organization, and gene-expression patterns [18–20]. Our findings are consistent with these observations and suggest that repeated vitrification-warming exposure may cumulatively impair embryo developmental competence, even when immediate post-warming survival remains acceptable. Exploratory age-stratified analyses further suggested that these adverse laboratory trends appeared numerically greater in women aged ≥ 38 years, particularly with respect to blastocyst developmental competence and euploid embryo yield per cleavage-stage embryo. This observation is biologically plausible, because reproductive aging may increase susceptibility to cryodamage through impaired mitochondrial dynamics, diminished cellular repair capacity, and altered autophagic regulation [21]. However, formal interaction testing did not demonstrate statistically significant vitrification-by-age interactions. Therefore, these subgroup findings should be interpreted as exploratory rather than evidence of confirmed age-dependent effect modification.

The present study observed reduced blastocyst formation, high-quality blastocyst rates, and euploid embryo yield per cleavage-stage embryo after repeated vitrification, whereas the euploid rate per biopsied blastocyst did not differ significantly between groups. Complementary embryo-level generalized linear mixed modeling likewise demonstrated a directionally lower adjusted probability of euploid embryo acquisition after repeated vitrification, although statistical significance was not reached. Taken together, these findings suggest that repeated vitrification may be more strongly associated with impaired embryo developmental progression than with detectable alterations in chromosomal constitution among blastocysts surviving to biopsy.

A similar pattern has also been reported in studies of oocyte vitrification, in which cryopreservation exposure reduced blastocyst formation, while euploid rates among biopsied blastocysts remained relatively preserved [22]. One possible explanation is selective developmental attrition (“survival of the fittest”), whereby embryos that are more vulnerable to cryopreservation-related stress fail during post-warming culture, whereas embryos with greater developmental resilience may be more likely to progress to the blastocyst stage [14, 23]. Consistent with this interpretation, previous studies have shown that euploid embryos are substantially more likely to progress to the blastocyst stage than aneuploid embryos [24], indicating that blastocyst development itself may represent a biologically selective process influenced by chromosomal status.

In addition, human embryos have been hypothesized to possess a limited capacity for developmental self-correction through the elimination or expulsion of abnormal blastomeres during cleavage and blastocyst development [25], which might be a potential explanation for the relative preservation of euploid blastocyst proportions despite developmental attrition during extended culture. Another possible explanation is that repeated vitrification exerts predominantly sublethal physiologic effects rather than directly inducing overt chromosomal abnormalities. Experimental evidence suggests that cryopreservation-related stress may impair mitochondrial activity, cellular metabolism, cytoskeletal integrity, or transcriptional regulation without necessarily causing detectable whole-chromosome aneuploidy. Such sublethal injury may, therefore, compromise embryo developmental efficiency while allowing surviving blastocysts to retain detectable euploid status [19].

A recent meta-analysis by Bickendorf et al. [10] reported significantly reduced live birth or ongoing pregnancy rates together with increased miscarriage rates after single-biopsy double-vitrification strategies. These findings differ from the statistically nonsignificant reproductive comparisons observed in our cohort. However, given the relatively limited transfer sample size and low detectable statistical power of the present study, our nonsignificant reproductive findings should be interpreted as inconclusive rather than reassuringly equivalent. In addition, differences in patient populations, indications for repeated vitrification, embryo selection strategies, and analytic denominators across studies may also contribute to heterogeneity in reported reproductive estimates.

From a clinical perspective, the present findings suggest that although repeated cryopreservation may still permit the acquisition of transferable euploid blastocysts, the additional vitrification-warming exposure at the cleavage stage may potentially reduce the number of transferable euploid embryos available per initiated treatment strategy, although the downstream impact on cumulative reproductive outcomes remains uncertain.

This study provides relatively comprehensive data spanning cleavage-stage embryo development, blastocyst genetic screening, and post-transfer clinical outcomes within a standardized embryo-pooling PGT-A framework, thereby offering a clearer assessment of repeated cryopreservation burden independent of re-biopsy-related confounding. Nevertheless, several limitations should be acknowledged. First, this was a retrospective single-center study, and residual confounding cannot be completely excluded. Second, mosaic embryos were not archived as an independent analytic category in the original PGT-A reporting framework and were instead incorporated into the overall non-euploid classification. Consequently, separate mosaic-specific analyses could not be performed, limiting the ability to determine whether repeated cryopreservation may differentially affect mosaic vs. uniformly aneuploid embryos. Because mosaic embryos may exhibit distinct developmental potential and implantation behavior, this classification approach may have influenced the precision of euploidy-related estimates and should, therefore, be recognized as an important limitation of the present retrospective dataset. Third, although no statistically significant differences were observed in pregnancy or neonatal outcomes, the available number of transfer cycles and live births remained limited, substantially limiting statistical power to exclude clinically meaningful reproductive differences between groups. Accordingly, the present clinical outcome analyses should be regarded as exploratory. Whether repeated vitrification-warming procedures induce cumulative long-term reproductive or offspring consequences warrants further investigation in larger prospective studies with extended follow-up.

## Conclusion

Repeated vitrification-warming exposure was associated with reduced embryo developmental efficiency, including lower blastocyst formation and high-quality blastocyst generation, together with reduced euploid embryo yield per cleavage-stage embryo. No statistically significant differences were observed in reproductive outcomes after euploid blastocyst transfer; however, the available clinical sample size was insufficient to exclude clinically meaningful downstream reproductive differences between groups. Therefore, the present findings should be interpreted cautiously, and larger, adequately powered studies are required to further clarify the reproductive implications of repeated cryopreservation.

## Supplementary Information

Below is the link to the electronic supplementary material.

Supplementary file1 (DOCX 103 kb)

## Data Availability

The data underlying this article are available from the corresponding author on reasonable request.
