# Supplementary material for: Impact of repeated cryopreservation on embryo development and chromosomal ploidy
Source: Arch Gynecol Obstet. 2026 Jun 2;313(1):242. doi: 10.1007/s00404-026-08475-3 (PMC13442481; doi:10.1007/s00404-026-08475-3)
Supplement: Supplementary file 1 [file 404_2026_8475_MOESM1_ESM.docx]

**Supplementary Materials**

Supplementary Table S1. Embryo-weighted crude laboratory rates derived from pooled event counts in the CV− and CV+ groups.

| Outcome | CV- group  n (%) | CV+ group  n (%) | Absolute between-group difference (%) |
| --- | --- | --- | --- |
| Blastocyst formation rate | 579/1281 (45.2) | 413/1054 (39.2) | −6.0 |
| High-quality blastocyst formation rate | 217/1281 (16.9) | 123/1054 (11.7) | −5.2 |
| High-quality blastocyst proportion | 217/579 (37.5) | 123/413 (29.8) | −7.7 |
| Euploid rate per biopsied blastocyst | 142/579 (24.5) | 88/413 (21.3) | −3.2 |
| Euploid rate per cleavage-stage embryo | 142/1281 (11.1) | 88/1054 (8.3) | −2.8 |

Crude embryo-weighted rates were calculated directly from pooled embryo event counts and are presented for descriptive comparison only. These descriptive estimates are not intended to replace the primary patient-clustered mixed-model analyses presented in Table 2.

Supplementary Table S2. Exploratory age-stratified subgroup analyses with corresponding tests for interaction.

| Outcome | Women <38 years, adjusted difference (95% CI) | *P-value* | Women ≥38 years, adjusted difference (95% CI) | *P-value* | *P* for interaction |
| --- | --- | --- | --- | --- | --- |
| Blastocyst formation rate | -8.5 (-20.5 to 3.4) | 0.164 | -6.6 (-11.6 to -1.7) | 0.009 | 0.760 |
| High-quality blastocyst formation rate | -8.8 (-20.6 to 3.0) | 0.146 | -4.9 (-8.2 to -1.5) | 0.005 | 0.413 |
| High-quality blastocyst proportion | -1.4 (-16.7 to 13.9) | 0.861 | -8.0 (-15.4 to -0.6) | 0.036 | 0.445 |
| Euploid rate per biopsied blastocyst | 0.4 (-14.5 to 15.3) | 0.957 | -3.7 (-8.9 to 1.5) | 0.166 | 0.531 |
| Euploid rate per cleavage-stage embryo | -3.9 (-14.2 to 6.3) | 0.454 | -2.5 (-4.8 to -0.2) | 0.036 | 0.680 |

Adjusted effects represent age-adjusted mean between-group differences estimated using the same cluster-robust mixed-model framework as in the primary analyses. Formal interaction testing between vitrification group and age stratum was additionally performed for each endpoint.

Supplementary Table S3 Sensitivity analysis of embryologic and euploidy-related outcomes after exclusion of embryos lacking complete laboratory follow-up.

| Outcome | CV- group (Mean ± SD) | CV+ group (Mean ± SD) | Crude difference (95% CI) | Crude *P*-value | Adjusted difference* (95% CI) | Adjusted *P*-value* |
| --- | --- | --- | --- | --- | --- | --- |
| Blastocyst formation rate, % | 46.7 ± 33.3 | 41.1 ± 39.3 | -5.6(-11.0 to -0.3) | 0.038 | -5.7(-11.0 to -0.4) | 0.035 |
| High-quality blastocyst formation rate, % | 17.9 ± 25.9 | 11.3 ± 25.1 | -6.6(-10.4 to -2.8) | 0.001 | -6.6(-10.4 to -2.9) | 0.001 |
| High-quality blastocyst proportion, % | 38.2 ± 40.4 | 28.2 ± 41.2 | -9.9(-17.5 to -2.3) | 0.011 | -10.3(-17.8 to -2.7) | 0.008 |
| Euploid rate per biopsied blastocyst, % | 23.3 ± 32.5 | 19.4 ± 34.8 | -3.9(-9.7 to 2.0) | 0.197 | -4.7(-10.4 to 1.0) | 0.107 |
| Euploid rate per cleavage-stage embryo, % | 11.4 ± 20.4 | 8.3 ± 21.1 | -3.2(-6.1 to -0.2) | 0.035 | -3.2(-6.1 to -0.3) | 0.031 |

This sensitivity analysis excluded embryos transferred in fresh cycles or discarded because of poor quality before blastocyst culture and included 597 cycles (CV− group: n = 236; CV+ group: n = 361), comprising 715 biopsied blastocysts and 172 euploid embryos.

Supplementary Figure S1 Sensitivity-analysis forest plots comparing embryologic and euploidy-related outcomes between the CV+ and CV− groups after exclusion of embryos lacking complete laboratory follow-up.

Data are presented as adjusted mean differences with 95% confidence intervals (CIs) derived from linear mixed models with cluster-robust standard errors. Figure S1A shows the overall analysis adjusted for female age at retrieval. Figure S1B shows exploratory age-stratified analyses, with blue squares representing women aged <38 years and red diamonds representing women aged ≥38 years. Error bars crossing the vertical reference line indicate no statistically significant between-group difference (*P* ≥ 0.05).

Supplementary Table S4. Complementary embryo-level generalized linear mixed-model analyses of euploid embryo probability.

| Cohort | Adjusted OR (95% CI) | *P-value* |
| --- | --- | --- |
| Primary analysis (all embryos) | 0.72 (0.48–1.07) | 0.101 |
| Sensitivity analysis (complete cycles only) | 0.69 (0.45–1.07) | 0.099 |

Generalized linear mixed models with binomial distribution were fitted using euploid embryo status as the binary outcome and patient as a random intercept. Models were adjusted for female age, stimulation protocol, indication, gonadotropin duration, gonadotropin dose, and cycle number.

Supplementary Table S5. Detectable effect-size context under the observed sample sizes for principal euploidy-related and reproductive outcomes.

| Outcome | CV- group  n (%) | CV+ group  n (%) | Absolute between-group difference (%) | *P-value* | Approximate detectable power under observed effect size (%) | Minimum detectable difference at 80% power (%) |
| --- | --- | --- | --- | --- | --- | --- |
| Euploid rate per biopsied blastocyst | 142/579 (24.5) | 88/413 (21.3) | 3.2 | 0.175 | 22.3 | 7.6 |
| Euploid rate per cleavage-stage embryo | 142/1281 (11.1) | 88/1054 (8.3) | 2.7 | 0.043 | 60.9 | 3.5 |
| Clinical pregnancy rate | 53/94 (56.4) | 32/54 (59.3) | 2.9 | 0.733 | 6.3 | 23.7 |
| Live birth rate | 48/94 (51.1) | 29/54 (53.7) | 2.6 | 0.757 | 6.1 | 23.9 |

These analyses were performed to provide quantitative context for interpreting statistically nonsignificant findings under the observed sample sizes and event numbers.
